# *Bifidobacterium animalis* ssp. Lactis 420 Mitigates Autoimmune Hepatitis Through Regulating Intestinal Barrier and Liver Immune Cells

**DOI:** 10.3389/fimmu.2020.569104

**Published:** 2020-10-06

**Authors:** Hongxia Zhang, Man Liu, Xin Liu, Weilong Zhong, Yanni Li, Ying Ran, Liping Guo, Xu Chen, Jingwen Zhao, Bangmao Wang, Lu Zhou

**Affiliations:** ^1^Department of Gastroenterology and Hepatology, General Hospital, Tianjin Medical University, Tianjin, China; ^2^Department of Gastroenterology and Hepatology, People's Hospital of Hetian District, Xinjiang Uygur Autonomous Region, China

**Keywords:** autoimmune hepatitis, *Bifidobacterium*, intestinal barrier, macrophages, Th17 cells, gut–liver axis

## Abstract

Autoimmune hepatitis (AIH) is an immune-mediated inflammatory liver disease of uncertain cause. Accumulating evidence shows that gut microbiota and intestinal barrier play significant roles in AIH thus the gut–liver axis has important clinical significance as a potential therapeutic target. In the present study, we found that *Bifidobacterium animalis* ssp. lactis 420 (B420) significantly alleviated S100-induced experimental autoimmune hepatitis (EAH) and modulated the gut microbiota composition. While the analysis of clinical specimens revealed that the fecal SCFA quantities were decreased in AIH patients, and B420 increased the cecal SCFA quantities in EAH mice. Remarkably, B420 application improved intestinal barrier function through upregulation of tight junction proteins in both vitro and vivo experiments. Moreover, B420 decreased the serum endotoxin level and suppressed the RIP3 signaling pathway of liver macrophages in EAH mice thus regulated the proliferation of Th17 cells. Nevertheless, the inhibition effect of B420 on RIP3 signaling pathway was blunted *in vitro* studies. Together, our results showed that early intervention with B420 contributed to improve the liver immune homeostasis and liver injury in EAH mice, which might be partly due to the protection of intestinal barrier. Our study suggested the potential efficacy of probiotics application against AIH and the promising therapeutic strategies targeting gut–liver axis for AIH.

## Introduction

Autoimmune hepatitis (AIH) is a chronic inflammatory liver disease with increasing incidence, while the underlying mechanisms remain unclear (1). In addition to genetic factors, various environmental factors have been implicated in the development of liver diseases (2–4). Recently, the gut microbiota has been recognized as a major environmental risk factor for AIH, and the associated mechanisms include disruption of the intestinal barrier, intestinal bacterial translocation, and break of immune tolerance (5, 6).

Recently, intestinal dysbiosis was reported in patients with AIH. In a Chinese cohort, disease-associated dysbiosis in steroid treatment-naïve AIH patients was characterized by reduced biodiversity and decreased abundance of anaerobes (6). Furthermore, Timur Liwinski et al. reported a disease-specific decline of the relative abundance of *Bifidobacterium* in patients with AIH (7), which suggested that probiotics might potentially exhibit a beneficial effect in AIH. *Bifidobacterium* is one of the most important bacterial groups found in the human intestinal tract and its characteristics and mechanism of action have been reported since 1950 (8–10). Moreover, *Bifidobacterium* has been clinically in some chronic diseases used to maintain the balance of intestinal microbiota without serious side effects (11–13). *Bifidobacterium animalis* subsp. lactis 420 (B420), known for its immunoregulatory properties and improving intestinal epithelial integrity in mice models, has been given to humans in earlier clinical trials (14–17). Therefore, we used B420 to explore the potential effects and application of probiotics in AIH in our experiment.

Short-chain fatty acids (SCFAs), primarily acetate, propionate, and butyrate, are the major products of the colonic microbial fermentation of undigested dietary fiber (18, 19). A double-blind and randomized clinical trial performed by Livia et al. proved that the intake of fermented milk containing *Lactobacillus* and *Bifidobacterium* seems to increase fecal SCFA (20). In particular, butyrate has potential immunoregulation properties and serves as the preferred metabolic substrate for intestinal epithelial cells (21, 22). Lipopolysaccharide (LPS), which is the major component of the outer membrane of most Gram-negative bacteria and is referred to as an endotoxin, plays a key role in gut–liver axis (23, 24). Interestingly, previous study found that SCFA could inhibit LPS-induced inflammatory responses, which indicated that SCFA might be an important protective metabolite in gut–liver interactions (25).

It is commonly accepted that macrophages are implicated in the pathological inflammation and fibrosis of liver diseases and activated macrophages are present in the portal area of AIH (26–28). Our previous studies have found that receptor-interacting protein kinase 3 (RIP3) signaling was involved in LPS-induced macrophage/monocyte activation in AIH (29). RIP3 kinase activity supports the recruitment of the mixed lineage kinase domain-like (MLKL) to trigger membrane leakage with the consequent production of pro-inflammatory cytokines (1, 30, 31). The composition of the local cytokine milieu dictates CD4+ Th cells to differentiate into specific T cell subsets, of which the Th17 cells are the main effector cells executing intensify inflammation and tissue injury functions in the live tissue of AIH (1, 32). Taken together, these findings indicated that environmental factors, especially intestinal microbiota, may involve in the activation of immune cells and loss of self-tolerance to autoantigens in persons genetically susceptible to AIH.

In the present study, we addressed the efficacy and associated mechanisms of probiotics on immune-mediated liver injury through B420 supplement. Our results showed that B420 alleviated liver injury in EAH mice, partly by modulating gut microbiota and RIP3 signaling of liver macrophages, and these effects were accompanied by the increase of cecal SCFA production, upregulation of intestinal tight junction proteins, repression of liver pro-inflammatory cytokines and a decrease of Th17 cells in liver and spleen. Collectively, these findings revealed that probiotics supplement might exhibit potential efficacy against AIH through targeting gut–liver axis.

## Materials and Methods

### Ethical Approval Statement

All experimental procedures were performed according to the guidelines of the Institutional Animal Care and Use Committee at Tianjin Medical University and followed the International Association of Veterinary Editors guidelines for the Care and Use of Laboratory Animal. The animal use protocol listed below has been reviewed and approved by the Animal Ethical and Welfare Committee of Tianjin Medical University, Approval No. IRB2015-YX-009.

### Animal Experiments

**Figure 1:**
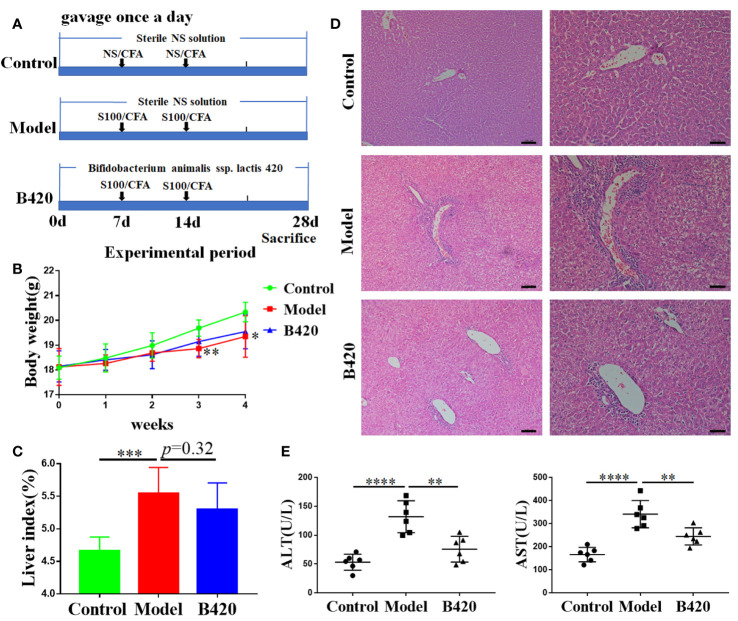
B420 attenuated liver injury in EAH mice. **(A)** Modeling process of EAH and administration of B420. **(B)** Body weight of each group was recorded weekly. **(C)** The liver index (liver wight/body weigh) between groups was measured. **(D)** Representative H&E images of liver tissues were shown (Scale bar: left:100μm, right:50μm). **(E)** The serum concentrations of ALT and AST were assessed. In **(A–D)**, n = 6 in each group. The data were presented as means ± SD (Student’s t-test, **p < 0.01, ***p < 0.001, ****p < 0.0001).

Twenty-six female SPF C57BL/6 mice (6 weeks of age) were purchased from Beijing Animal Study Centre, and maintained under specific pathogen-free conditions in Animal Centre of the Tianjin Medical University. All mice were randomly divided into three groups including control group (n=6), model group(n=6) and B420 group(n=6). The rest mice (n=8) were killed and hepatic antigen S100 were extracted after perfusion of livers with cold phosphate-buffered saline (PBS) as previous description (33). Briefly, the livers were cut into scrap and homogenized with cold PBS on ice. After ultrasonic grinding, the homogenate was centrifuged at 150 g for 10 min to remove nuclei. Next, the supernatants were centrifuged at 100,000 g for 1 h. The supernatants further ran through a 90-cm CL-6B Sepharose column (Pharmacia, Freiburg). The first nontoxic peak was acquired and concentrated to 0.5–2.0 g/L. The model group and B420 group were intraperitoneal immunized with 0.5 ml liver S100 antigen emulsified in an equal volume of complete Freund’s adjuvant (CFA, sigma, USA) on day 7 and day 14 to induce experimental autoimmune hepatitis (EAH) and the control group was intraperitoneal injected with 0.5 ml sterile normal saline (NS) with an equal volume of CFA on day 7 and day 14. Mice of B420 group were treated with *B. animalis* ssp. lactis 420 (B420) (DuPont Nutrition & Biosciences, China; ATCC: SD6685, 10^9^ CFU/200ul) dissolved in sterile NS *via* gavage and mice of the other two groups were gavaged 200ul NS every day for 4 weeks. On day 28, all the animals were sacrificed under anesthesia (**Figure 1A**).

### Participants

A total of fourteen AIH patients and six controls were included. AIH patients were recruited from the Gastroenterology Department at Tianjin Medical University General Hospital. The diagnosis of AIH was made if patients conformed with (1) 1999 revised International Autoimmune Hepatitis Group (IAIHG) score ≥10 and/or (2) 2008 IAIHG simplified AIH score ≥6 and/or (3) histological features indicative of AIH (34, 35). All the patients were collected before corticosteroids therapy. Control subjects were selected from the health manage center of Tianjin Medical University General Hospital to match with AIH patients for age and gender. The control group had to fulfil the following inclusion criteria: (1) normal ranges of liver function test, (2) an absence of hepatitis B/C virus antigen, (3) normal abdominal ultrasound test, (4) an absence of autoimmune diseases and family history. The feces of the individuals were collected.

### Liver and Ileum Histological Examination

Liver tissues and ileum tissues were collected and fixed in 4% paraformaldehyde after mice were sacrificed. The paraffin embedded liver tissues and intestinal tissue were sectioned at approximately 5µm and processed for staining with hematoxylin and eosin (H&E) according to the standard H&E protocol. The pathological change of the liver and intestinal tissue was evaluated by two independent and experienced pathologists. The liver histopathology index was measured according to the Ishak system including periportal interface hepatitis, confluent necrosis, focal lytic necrosis and portal inflammation (36).

### Biochemical Analysis and Enzyme-Linked Immunosorbent Assay (ELISA)

The blood of the mice was centrifuged at 150 g for 10 min. The serum was then stored at −80°C. The serum concentration of LPS was quantified with the ELISA kits according to the manufacturer’s instructions (eBioscience). The serum alanine aminotransferase (ALT) and aspartate aminotransferase (AST) levels were tested by using the automated chemistry analyzer (AU5800, Beckman Coulter, USA) from the clinical laboratory of the Tianjin Medical University General hospital.

### Intestinal Microbiota Analysis

The 16S rRNA gene sequencing procedure was performed by the GENEWIZ Genomics Institute (Suzhou, China). Total fecal bacteria DNA extractions were acquired from cecal specimens of each 3-week old and 8-week old offspring by QIAamp ^®^ Fast DNA Stool Mini Kit (QIAamp, Germany). The microbial 16S V3-V4 region was amplified with indexes and adaptors-linked universal primers (341F: ACTCCTACGGGAGGCAGCAG, 806R: GGACTACHVGGGTWTCTAAT). PCR was performed using KAPA HiFi Hotstart PCR kit high fidelity enzyme in triplicate. Amplicon libraries were quantified by Qubit 2.0 Fluorometer (Thermo Fisher Scientific, Waltham, US) and then sequenced on Illumina HiSeq platform (Illumina, San Diego, US) for paired-end reads of 250 bp. After discarding the singletons and removing chimeras, tags were clustered into operational taxonomic units (OTUs) using USEARCH (v7.0.1090) at 97% similarity. Afterwards, a representative sequence of each OTU was subjected to the taxonomy-based analysis using the RDP database. Heatmap was created using R. Cluster analysis. Alpha diversity and beta diversity were analyzed using QIIME. The relative abundance of bacteria was expressed as the percentage.

### Cecal Short-Chain Fatty Acid Quantification

The SCFA concentrations were determined by gas chromatography (GC) as previously described (37). Briefly, the feces from participants and cecal contents from mice were diluted, acidified, and extracted ultrasonically on ice for 10 min. The samples were then centrifuged at 12,000 g and 4°C for 15 min. After the supernatant was mixed with ethyl acetate (1:1), the extract was filtered through a 0.22-µm pore-size filter and poured into an Agilent 7890A Series GC. The SCFA standards were purchased from Sigma-Aldrich (St. Louis, MO, USA).

### *In Vivo* Permeability Assay

Intestinal permeability was determined by FITC-dextran assay. FITC-D (4000 MW, Sigma-Aldrich) dissolved in normal saline infusion (50 mg/mL) and was administrated to the mice by gavage at 6 mg/10 g body weight. Whole blood was collected 4 h after FITC-D administration using heparinized microhematocrit capillary tubes *via* eye bleed. Sera was extracted from the blood by centrifuging at 4°C for 10 min at 2,000 rpm. Fluorescence intensity analysis was carried out using a plate reader. The concentration of FITC-D of each mouse was detected based on the FITC-D standard curve.

### Quantitative Realtime PCR (qRT-PCR)

Total RNA was extracted using the RNeasy mini kit (Qiagen, Carlsbad, CA, USA) followed by cDNA reverse transcription using the TIANScript RT Kit (TIANGEN, Inc. Beijing, China) according to the manufacturer’s protocol. Realtime-PCR analysis was performed using Taqman Gene Expression Master Mix and primes (GENEWIZ, Inc. Beijing, China). The Oligonucleotide primers for target genes were listed in **Table 1**. Glyceraldehyde-3-phosphate dehydrogenase (GAPDH) was employed as an endogenous control. The relative mRNA expression levels of the target gene were evaluated by calculating the fold-changes normalized to the GAPDH for each sample using 2^−ΔΔCt^ method. All cDNA samples were analyzed in triplicate.

**Table 1:** The Oligonucleotide primers used in realtime-PCR analysis.

Murine gene	Primer sequences (5′- 3′)
GAPDH ZO-1 Occludin TNF-α IL-6 IL-1β RIP3 MLKL CCL2 CCR2	Forward primer: TGTGTCCGTCGTGGATCTGA Reverse primer: CCTGCTTCACCACCTTCTTGA Forward primer: GGGCCATCTCAACTCCTGTA Reverse primer: AGAAGGGCTGACGGGTAAAT Forward primer: ACTATGCGGAAAGAGTTGACAG Reverse primer: GTCATCCACACTCAAGGTCAG Forward primer: ACTCCAGGCGGTGCCTATG Reverse primer: GAGCGTGGTGGCCCCT Forward primer: CCAGTTGCCTTCTTGGGACT Reverse primer: GGTCTGTTGGGAGTGGTATCC Forward primer: GTGGCTGTGGAGAAGCTGTG Reverse primer: GAAGGTCCACGGGAAAGACAC Forward primer: GAAGACACGGCACTCCTTGGTA Reverse primer: CTTGAGGCAGTAGTTCTTGGTGG Forward primer: CCTTGCTTGCTTGCTTTT Reverse primer: TTTCCTTGAGTTTGAGCCA Forward primer: ACCTTTTCCACAACCACCT Reverse primer: GCATCACAGTCCGAGTCA Forward primer: AAGGGTCACAGGATTAGGAAG Reverse primer: ATGGTTCAGTCACGGCATA

### Western Blotting

The liver and intestinal tissues were dissolved in RIPA buffer with protease inhibitors (Solarbio, Beijing, China). After homogenization, the protein concentrations were determined by Bicinchoninic acid protein assay (Thermo Scientific Inc). Proteins were separated using SDS-polyacrylamide gel electrophoresis system and then blotted onto a polyvinylidene fluoride (PVDF) membrane (Invitrogen, Carlsbad, CA, USA). Afterwards, the primary anti-RIP3 (ab62344, Abcam, Cambridge, MA, USA), anti-MLKL (ab196436, Abcam, Cambridge, MA, USA), anti-ZO-1 (ab96587, Abcam, Cambridge, MA, USA), anti-Occludin (ab216327, Abcam, Cambridge, MA, USA), and anti-GAPDH (rabbit, antimouse, Cell Signaling Technology)antibody were applied; anti-GAPDH antibody was employed as the loading control. After incubated with horseradish peroxidase (HRP)–conjugated secondary antibodies (Cell Signaling Technology), the chemiluminescent signal was detected. The intensity of the band was determined by image processor program (Image J).

### Immunofluorescence

Immunofluorescence analyses for tight junction proteins of the mice intestinal tissue were performed were performed with 4mm-thick frozen sections. Slides were fixed with acetone and blocked with 5% bovine serum albumin then incubated with antibodies against ZO-1 (Abcam, USA) or Occludin (Abcam, USA) overnight at 4°C. Subsequently, the sections were washed with PBS for 5 min three times and incubated with Alexa Fluor 488 (Santa Cruz Biotechnology, Inc) at room temperature in the dark for 60 min. Nuclear staining was achieved by 4’, 6-diamidino-2-phenylindol (DAPI). Double immunofluorescence analyses for macrophage of liver tissue were performed as shown above. Slides were fixed with acetone and blocked with 5% bovine serum albumin then incubated with antibodies against F4/80 (ab16911, Abcam, Cambridge, MA, USA) and RIP3 (Abcam, USA), or F4/80 (Abcam, USA) and MLKL (Abcam, USA), further incubated with Alexa Fluor 488 (Santa Cruz Biotechnology, Inc) and Alexa Fluor 568 antibody (Santa Cruz Biotechnology, Inc). DAPI was lastly applied to dye the nucleus. Fluorescence photographs were obtained under fluorescence microscope DM5000 B (Leika, Germany).

### Cell Isolation and Flow Cytometry Analysis

Single-cell suspensions of lymphocyte were harvested from spleen and liver of mice. Prior to intracellular cytokine staining, cells were stimulated with PMA and ionomycin (BD Bioscience) in the presence of brefeldin A (BD Bioscience) for 5h. Cells were collected, washed by PBS, and stained with APC-conjugated anti-mouse CD4 antibody in the presence of FcR-Block (BD Bioscience) in dark for 30 min. After the wash, cells were fixed by CytoFix/Cyto Perm buffer (BD Bioscience) and stained with PE-conjugated anti-mouse IL-17A (BD Bioscience) anti-body or isotype control antibody for 30 min. Data were obtained on a FACS Calibur (BD Bioscience) and analyzed using FlowJo 7.6 software.

### Bacterial Culture and Bifidobacterium Supernatants

B420 was supplied by DuPont Nutrition & Biosciences and incubated in brain heart infusion medium under anaerobic conditions for 24 h at 37°C until the logarithmic phase of growth with a bacterial density of 0.5 at optical density (OD) 600. The culture suspensions were centrifuged at 5,000 × g for 10 min at 4°C, then the supernatant (B420-s) was collected and filter-sterilized through 0.22 μm filters. The B420-s was diluted to three concentration gradients (1:100, 1:50,1:20) with complete culture medium.

### Cell Cultures

The mouse macrophage cell line RAW264.7 (ATCC SC-6003) was cultured in a Dulbecco modified Eagle medium (DMEM) (Gibco) in 10% fetal bovine serum (FBS) (Gibco), 50 U/ml penicillin and 50 U/ml streptomycin (all from Invitrogen, USA) in a humidified incubator containing 5% CO2 at 37°C. RAW264.7 cells were seeded in a 12-well plate at a density of 1×10^5^ cells per well. Human Caco-2 cells (BNCC 338148) were cultured in Modified Eagle’s Medium (MEM) (Gibco) supplemented with 20% fetal bovine serum and a penicillin-streptomycin solution. The cells were incubated under same conditions as above and were seeded in a 12-well plate at a density of 1×105 cells per well. In the LPS experiments (LPS group), the cells were treated with LPS (3mg/ml, Solarbio Biotech) for 12 h. In the (B420-s) experiments (LPS-B420-s group), the cells were pre-treated with B420-s of three concentration gradients (1:100, 1:50,1:20) for 3h, then treated with LPS (3 mg/ml) for 12 h.

### Statistical Analysis

Data were presented as the mean ± SD. The statistical significance of differences was assayed by one-way ANOVA in multiple groups, and t-tests for paired samples using SPSS 22.0 (SPSS, Chicago, IL, USA). All the differences were considered as statistically significant at p < 0.05.

## Results

### B420 Attenuated Liver Injury in EAH Mice

Previous research found that a reduced number of fecal anaerobes, represented by the disease-specific decline of *Bifidobacterium*, occurred in AIH patients (7, 38). Our preliminary study found that early intervention of B420 could alleviate liver injury in concanavalin A (Con A) induced hepatitis mice model and decrease the expression of proinflammatory cytokines in the liver tissues (**Supplementary Figures 1A–C**). To further explore the therapeutic potentials and mechanism of probiotics on AIH, we gavage the EAH mice with B420 (**Figure 1A**). In this study, we found that the mice in the model group had decreased body weight at 3–4 weeks of the modeling process, with no significant difference between model group and B420 group (**Figure 1B**).

The general view of liver was shown in **Supplementary Figure 2A**. The liver index was significantly increased in the model group compared to the control group, but there was no statistical difference observed between the model and B420 groups (**Figure 1C**). Significantly, the representative images of liver tissue (as indicated by H&E staining) showed that the model group had severe infiltration of inflammation cells in the portal area and B420 supplement alleviated liver inflammation (**Figure 1D**). Similarly, we also evaluated the general view of spleen and spleen index and results showed that the model group had significantly increased spleen index compared to the control group, and B420 treatment decreased the spleen index (**Supplementary Figures 2A, B****)**. Accordingly, the model group had higher alanine transaminase (ALT) and aspartic transaminase (AST) levels compared to control group and B420 supplement significantly decreased the transaminase levels (**Figure 1E**). Together these findings indicated that B420 supplement in early stage of AIH contributed to attenuate the infiltration of inflammatory cells and liver injury in EAH mice.

### B420 Altered Composition and Diversity of Gut Microbiota in EAH Mice

**Figure 2:**
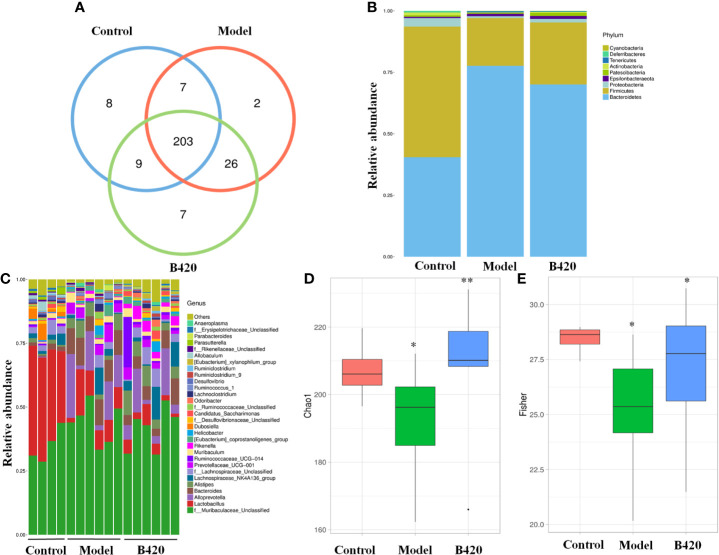
B420 altered the composition and diversity of gut microbiota in EAH mice. Total fecal bacteria from each mouse were detected by 16S rRNA sequencing. **(A)**Venn diagram. **(B)** Relative abundance of bacterial taxa at the phylum level between groups. **(C)** Relative abundance of bacterial taxa at the genus level in each mouse. **(D, E)** Chao1 and Fisher diversity index were shown. In **(A–E)**, Control: n=4, Model and B420: n=6 (*p < 0.05, **p < 0.01).

Emerging findings demonstrated intestinal dysbiosis in autoimmune disease and alterations of intestinal microbiota, such as depletion of obligate anaerobes and expansion of potential pathobionts, have been reported in AIH patients (6, 38). To further explore the effect of B420 on gut–liver axis, we studied the fecal microbiomes of the mice. The comparison of the OTUs among the three groups revealed 227OTUs in the control group, 238 OTUs in the EAH group and 245 OTUs in the B420 group, and a total of 203 OTUs were shared by the three groups (**Figure 2A**). The gut microbiota of all the samples was dominated by three major phyla: Bacteroidetes, Firmicutes and Proteobacteria. Notably, compared to the control group, a higher abundance of Bacteroidetes and a lower abundance of Firmicutes and Proteobacteria were observed in model group, which resulted in a decreased Firmicutes/Bacteroidetes (F/B) ratio compared to that in the control group. However, B420 did not statistically affect the F/B ratio (**Figure 2B**). The genus-level analysis revealed that the model group had increased relative abundance of potential pathogenic bacteria, such as Bacteroides and Ruminococcus, whereas B420 weakened this increase. Additionally, a relatively lower abundance of *Lactobacillus* was observed in mice of model group compared to the control group, and B420 treatment restored the abundance of *Lactobacillus* (**Figure 2C**). The Chao1 and Fisher index revealed that the model group had significantly decreased alpha diversity compared to the control group and B420 treatment restored the two indices, which suggested that B420 exerted stronger positive effects on alpha diversity of the gut microbiota (**Figures 2D, E**).

**Figure 3:**
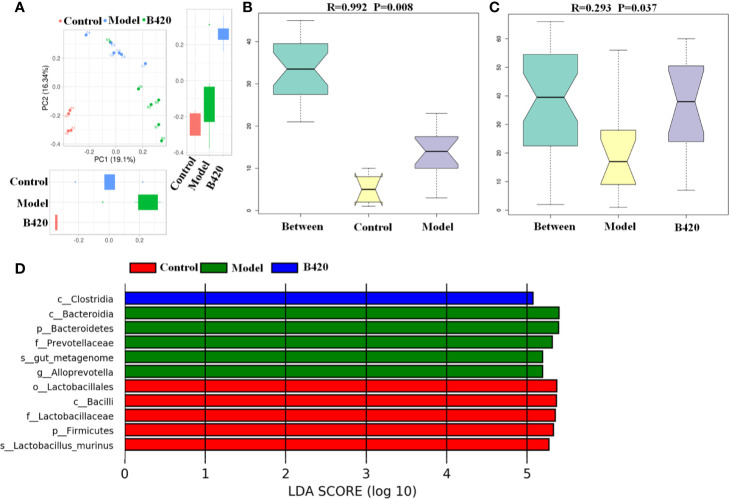
B420 altered the beta diversity of gut microbiota and the key bacterial alterations in mice. Total fecal bacteria from each mouse were detected by 16S rRNA sequencing. **(A)** Beta diversity. **(B, C)** Unweighted Unifrac ANOSIM analysis between **(B)** Control and Model group, **(C)** Model and B420 group. **(D)** Different abundant species at the phylum, class, order, family, and genus level generated by LEfSe analysis was shown. In **(A–D)**, Control: n=4, Model and B420: n=6.

A principal component analysis (PCA) based on weighted UniFrac distances revealed a different structure between the three groups (**Figure 3A**). To further quantify the differences in species diversity between groups, ANOSIM was projected and the results indicated the differences between groups were significant (**Figures 3B, C**). Furthermore, different abundant species among the three groups were examined by LDA EffectSize analysis. Results showed that the relative abundance of Alloprevotella and Prevotellaceae which were reported to be associated with rheumatoid arthritis (39), were higher in model group. Meanwhile, the potential pathogenic bacteria, such as Bacteroides and Ruminococcus, were also significantly increased in model group. The abundance of beneficial bacteria including Alistipes and Rikenella was significantly increased in B420 group. Importantly, clostridiales, associated with the production of SCFA, was abundant in the B420 group (**Figure 3D**).

Taken together, these results proved that EAH mice had a major alteration in the gut microbiota composition, whereas B420 at least partly altered the gut microbiota dysbiosis, which indicated the significance of probiotic supplement targeting gut–liver axis in maintaining immunological balance of the liver.

### B420 Increased the Level of Fecal SCFAs in EAH Mice

**Figure 4:**
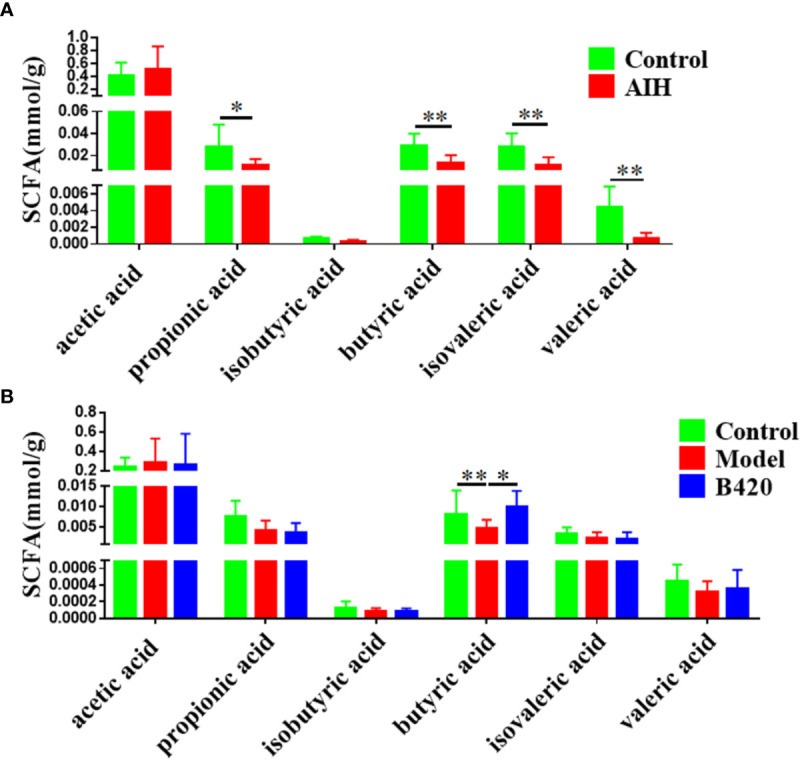
B420 increased the cecal butyric acid in EAH mice. **(A)** Alterations in the SCFA levels in the fecal contents of AIH patients. **(B)** Effect of B420 on SCFA in the cecal contents. In **(A)**, Control: n=6, AIH =14. In **(B)**, n=6 in each group. The data were presented as means ± SD (Student’s t-test, *p < 0.05, **p < 0.01).

SCFAs are major end-products of gut microbial fermentation and are implicated in the regulation of immune system and intestinal epithelial cells (40–42). In our study, the SCFA levels in the feces of AIH patients and controls were detected. The results showed that most abundant SCFAs in feces were acetic acids with less of butyric acids and propionic acids. Importantly, we found that there was a significant decrease of butyric acids as well as propionic acid, isovaleric acid and valeric acid in feces of AIH patients compared to controls (**Figure 4A**). Moreover, cecal feces from mice of the three groups were also collected and analyzed for the presence of SCFAs. Notably, the model group had significantly decreased concentration of butyric acids compared to the control group while B420 treatment increased the concentration of butyric acids (**Figure 4B**). Besides, we investigated the effect of butyrate on liver injury in Con A-mediated autoimmune hepatitis model (**Supplementary Figure 3A**). The results showed that butyrate could alleviate liver inflammation and decrease the transaminase levels (**Supplementary Figures 3B, C**). The butyrate group had lower expressions of RIP3 in the liver tissues compared to the Con A group as well as the expressions of IL-6 and IL-1β (**Supplementary Figures 3D, E**). These results suggest that the protective effects of probiotics therapy on autoimmune diseases might be partly due to alterations in microbial-derived metabolites.

### B420 Alleviated the Damage of Intestinal Barrier Function in EAH Mice

Intestinal barrier is essential for the maintenance of homeostasis in health and disease (43). A wealth of studies had shown that the intestinal barrier, part of the gut–liver axis, played a role in the pathophysiology of autoimmune diseases (44–47). Here, we explored whether the probiotic approach alleviated autoimmune liver injury through targeting impaired intestinal barrier function.

**Figure 5:**
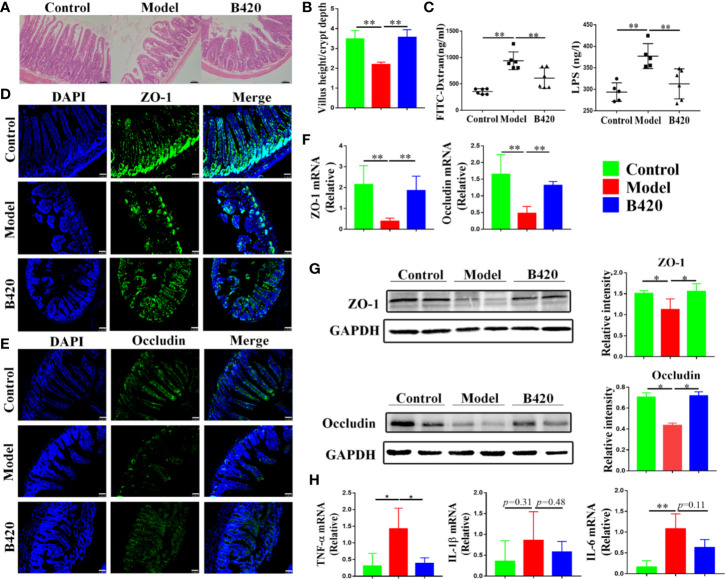
B420 alleviated the damage of intestinal barrier function in EAH mice. **(A)** H&E images of ileum tissues were shown. **(B)** Five crypts per section were evaluated, then microscopically assessed villus height, crypt depth and calculated the ratio of villus height and crypt depth. **(C)** Intestinal permeability was detected using the *in vivo* FITC-dextran assay and serum LPS testes. The FITC-dextran and LPS levels in serum is shown. **(D, E)** The membrane localization of **(D)** ZO-1 and **(E)** Occludin was assessed by immunostaining and visualized by fluorescence microscopy, nuclei were stained with DAPI (blue staining) and the green regions indicated ZO-1 and Occludin. **(F)** Total RNA was extracted from the ileum tissues for real-time PCR analysis. The relative expressions of ZO-1 and Occludin were shown. **(G)** Protein levels of ZO-1 and Occuludin in the ileum tissues from each group were detected by western blotting and the relative intensity was quantified. **(H)** The relative expressions of TNF-α, IL-6, and IL-1β in ileum tissues were analyzed. FITC-dextran, fluorescein isothiocyanate conjugated-dextran. In **(A–G)**, n = 6 in each group. Scale bar: 50μm. The data were presented as means ± SD (Student’s t-test, *p < 0.05, **p < 0.01).

**Figure 5A** showed the H&E staining of small intestines from the three groups. There was a damage of normal intestinal structure in mice of model group compared to that of control group. However, the B420 group showed lesser intestinal tract lesions compared to the model group. Further, the ratio of villus height to crypt depth was calculated to evaluate intestinal morphological alteration. The ratio in model group was significantly decreased compared to that in the control group, and B420 treatment restored the ratio back to that in the control group (**Figure 5B**). Next, the intestinal permeability and the integrity of the gut barrier were examined. The FITC-dextron and LPS tests showed that the mice in the model group had increased intestinal permeability and higher serum LPS levels compared to those in the control group, and it’s worth noting that B420 treatment significantly reduced intestinal permeability and alleviated endotoxemia (**Figure 5C**).

To further assess the integrity of intestinal barrier in these mice, we detected the structural proteins including zonula occludens-1 (ZO-1) and Occludin. Immunostaining of tight junction proteins suggested that the model group had reduced expressions of ZO-1 and Occludin in the small intestinal and B420 treatment increased the expressions of the structural proteins (**Figures 5D, E**). Remarkably, both the mRNA and protein expressions of ZO-1 and Occludin were significantly decreased in mice of model group compared to that in control group and B420 treatment upregulated the expressions of the structural proteins (**Figures 5F, G**). In addition, there was a significant increase in the expressions of TNF-α, IL-6 and IL-1β (barrier-disrupting cytokines) in the gut mucosa of the model group compared to that in the control group and B420 treatment significantly decreased the expression of TNF-α (**Figure 5H**).

Collectively, we addressed that there was intestinal barrier dysfunction accompanied by elevated levels of endotoxin in EAH model and early application of B420 could improve the intestinal barrier, which indicated a promising prospect of novel therapeutic strategies including probiotics and stabilization of tight junctions in AIH.

### B420 Inhibited the RIP3-MLKL Signaling Pathway of Liver Macrophages in EAH Mice

**Figure 6:**
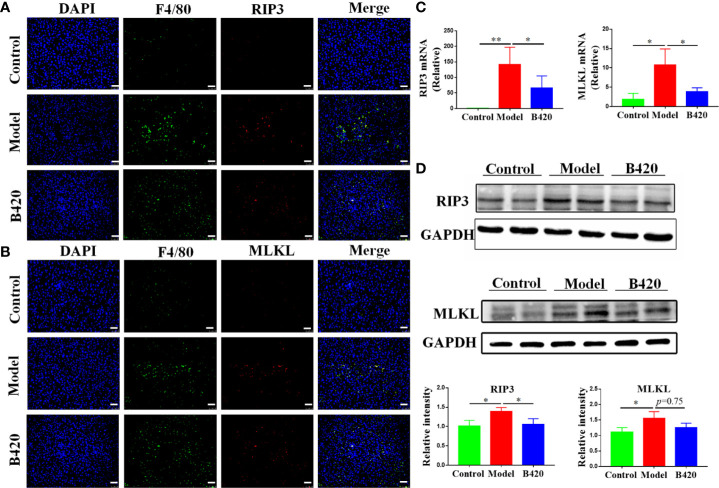
B420 inhibited the RIP3 signaling pathway of liver macrophages in EAH mice **(A)** Representative fluorescence images of liver tissues co-stained with F4/80 and RIP3. F4/80 (green), RIP3(red), DAPI (blue). **(B)** Representative fluorescence images of liver tissues co-stained with F4/80 and MLKL. F4/80 (green), MLKL (red), DAPI (blue). **(C)** Total RNA was extracted from the liver tissues for real-time PCR analysis. The relative expressions of RIP3 and MLKL were shown. **(D)** Protein levels of RIP3 and MLKL in the liver tissues from each group were detected by western blotting and the relative intensity was measured. In **(A–D)**, n=6 in each group. Scale bar: 50μm. The data were presented as means ± SD (Student’s t-test, *p < 0.05, **p < 0.01).

RIP3 has been increasingly recognized as a key inflammatory signal adapter, which mediates inflammation through necroptosis as well as non-necroptosis function (30, 48). Our previous studies have found that RIP3 signaling was involved in macrophage/monocyte activation in the liver tissues of AIH patients and is correlated with the levels of serum hepatic enzyme (29). Then, we examined the activation of RIP3 and MLKL (the direct downstream effector of RIP3) in liver macrophages of the mice. With anti-F4/80 Ab to identify Kupffer cells, RIP3 and MLKL were stained in the cells. As shown in **Figures 6A, B**, the majority of F4/80+ macrophages in the liver tissues of EAH mice expressed both RIP3 and MLKL. In contrast, the co-localization of F4/80+ macrophages and RIP3 or MLKL was rarely observed in the liver tissues of controls. Compared to the model group, the B420 group had a lower expression of RIP3 or MLKL in F4/80+ macrophages. Subsequently, the liver tissue of model group showed significantly increased expressions of both RIP3 and MLKL compared to the control group and the B420 group had lower expressions of RIP3 and MLKL (**Figure 6C**). The protein levels of RIP3 and MLKL were then detected by western blotting. As expected, our results showed that the protein levels of RIP3 and MLKL were significantly decreased in model group and the B420 group had a lower expression of RIP3, but the difference of MLKL between the model group and B420 group was not statistically significant (**Figure 6D**).

Thus, the downregulation of RIP3 signaling of liver macrophages might be a critical mechanism involved in immunoregulation and hepatoprotective effects of probiotics application in AIH.

### B420 Regulated Pro-Inflammation Cytokines and Chemokines in Liver as Well as Th17 Cells in Liver and Spleen

**Figure 7:**
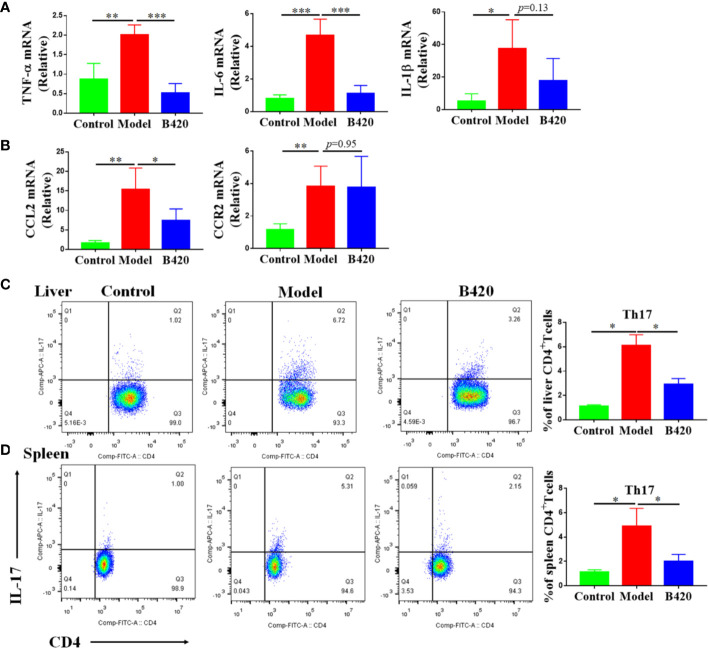
B420 regulated pro-inflammation cytokines and chemokines in liver as well as Th17 cells in liver and spleen. Total RNA was extracted from the liver tissues for real-time PCR analysis and mononuclear lymphocytes were isolated from liver and spleen, counted and stained with cell markers to identify Th17 cells. **(A)** The relative expressions of TNF-α, IL-6 and IL-1β in liver tissues were analyzed by real-time PCR. **(B)** The relative expressions of CCL2 and CCR2 mRNA in liver tissues were detected. **(C)** Typical CD4+IL-17+ Th17 cells flow cytometric plots and the percentage of Th17 cells out of the liver CD4+population were calculated. **(D)** Representative flow cytometry plots and the percentage of Th17 cells out of the spleen CD4+population were calculated. In **(A–D)**, n=6 in each group. The data were presented as means ± SD (Student’s t-test, *p < 0.05, **p < 0.01, ***p < 0.001).

To further explore the protective mechanism of B420 supplement in AIH, we also evaluated the expressions of liver inflammatory cytokines and chemokines. We found that the cytokine-secretion phenotype in the liver tissue of EAH mice was skewed towards M1-type macrophages leading to a highly inflammatory cytokine milieu enriched for TNF-α, IL-6 and IL-1β and B420 treatment significantly decreased the expressions of TNF-α and IL-6 **(****Figure 7A**). Besides, the expressions of chemokine ligand 2 (CCL2) and chemokine receptor type 2 (CCR2) also increased in model group compared to that in the control group and B420 significantly inhibited the expression of CCL2 (**Figure 7B**).

It is generally accepted that cytokine imbalance driven by increased pro-inflammation cytokine production of local innate immune responses favors CD4+ T cells responses and Th17 cells are effector cells that intensify inflammation and tissue injury (32, 49). Therefore, we measured the percentage of Th17 cells in the CD4 population of liver and spleen *via* flow cytometry. The results showed that the model group had significantly increased percentage of Th17 cells in both liver and spleen compared to the control group. Interestingly, B420 treatment counteracted this change (**Figures 7C, D**). All these data illustrated that B420 might regulate the local cytokine milieu thus affect the adaptive immune response, which contributed to improve the liver immune homeostasis and liver injury in AIH.

### Protective Effects of B420-s in LPS-Induced Barrier Injury of Caco-2 Monolayers and Activation of RAW264.7 Cells

**Figure 8:**
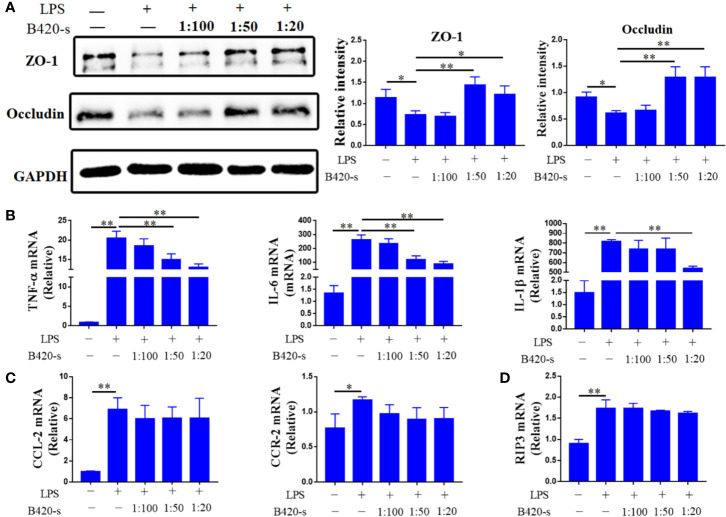
Protective effects of B420-s in LPS-induced barrier injury of Caco-2 monolayers and activation of RAW264.7 cells. The cells were treated with LPS (3 mg/ml) for 12 h in the absence or presence of pre-treatment with different concentrations of B420-s (1:100–1:20). **(A)** The relative protein expressions of ZO-1 and Occludin in Caco-2 cells were analyzed by western blotting. **(B–D)** TNF-α, IL-6 and IL-1β **(B)**, CCL2 and CCR2 **(C)**, RIP3 **(D)** mRNA levels in RAW24.7 cells were detected by quantitative real-time PCR. The data were presented as means ± SD of three independent experiments (Student’s t-test, *p < 0.05, **p < 0.01).

Our data indicated that B420 attenuated liver injury through improving intestinal barrier and regulated liver immune homeostasis in EAH mice. However, the link between intestinal barrier integrity and liver immune homeostasis is not clear. To investigate the effects of B420 on intestinal barrier function, we used a vitro model in which Caco-2 epithelial cell monolayers were treated with LPS as it has been demonstrated that LPS caused intestinal barrier dysfunction (50). The western blot results showed that the expressions of ZO-1 and Occludin were dramatically decreased in the LPS group compared to the control group. Whereas, the expressions of ZO-1 and Occludin were significantly increased in groups exposed to 1:50 B420-s or 1:20 B420-s compared to that in the LPS group (**Figure 8A**).

Next, the effects of B420-s on mRNA expressions of various proinflammatory cytokines in RAW264.7 cells were determined by quantitative real-time PCR under LPS-stimulated conditions. As shown in **Figure 8B**, the expressions of TNF-α, IL-6 and IL-1β were significantly increased in LPS-stimulated group compared to that in the control group. In the case of exposure to 1:50 B420-s, the expressions of TNF-α and IL-6 were significantly inhibited, but the expression of IL-1β was not inhibited unless the concentration of B420-s increased to 1:20. Exposure to LPS also increased expressions of CCL2 and CCR2 to a level significantly higher than that in the control group. However, when macrophages were exposed to different concentrations of B420-s combined with LPS, the expressions of CCL2 and CCR2 were not significantly inhibited and RIP3 wasn’t inhibited either (**Figures 8C, D**). Further, we used inactive B420-s to stimulate the Caco2 cells and Raw264.7 cells and the results showed that inactivate B420-s had no protective effect on LPS-induced barrier injury and activation of macrophages, which indicated that the direct immunoregulatory and protection of intestinal barrier effects of B420 (**Supplementary Figures 4A–D**). Together, the results of *in vitro* experiments indicated that the immunoregulatory effect of B420 in liver might be partly due to the protection of intestinal barrier.

## Discussion

The incidence of AIH is rising and has become an important cause of cirrhosis but the pathogenesis has not been completely explained (1). Recently, environmental factors, especially intestinal dysbiosis and impaired gut barrier function are considered to be associated with the development of AIH (38, 51). The restoration of an altered gut microbiota using probiotics is considered a potential strategy for the prevention and treatment of autoimmune diseases (52, 53). It has been proven that probiotics have beneficial integral functions in many chronic diseases, including inflammatory bowel disease, diabetes and obesity (54, 55). However, few trials have been performed using probiotics in AIH. One of the first colonizers of the human gut, Bifidobacterium, has been well studied for its effect on modulation of intestinal barrier function and SCFA metabolites (56), as well as for its critical role in controlling the immunoregulatory response (57). In our study, we try to explore whether probiotics supplement can alleviate liver injury and its underlying effect on gut–liver axis in EAH model.

It is commonly accepted that gut dysbiosis is associated with AIH and influenced diseases activity (6). However, the effect of therapy targeting intestinal microbiota in AIH remains obscure. The genus Bifidobacterium is one of the most well studied and widely applied probiotic bacteria, especially in the modulation of gut microbiota (58). As reported by Andrea et al., *Bifidobacterium* strains could restore the gut microbial balance in coeliac children as well as re-establishment of the physiological F/B ratio (59). In our study, although inter-animal variance existed in B420 group, we found that B420 significantly increased the alpha diversity of the gut microbiota and altered the composition of EAH mice characterized with the reduction of Bacteroides and Ruminococcus and increasing of *Lactobacillus*, Alistipes and Rikenella at the genus levels. Additionally, B420 treatment increased the abundance of Clostridia which is associated with butyric acid production. Along this line, our data revealed that early intervention with B420 can alter the diversity and composition of microbiota in EAH mice, even though there is an inter-animal variance within the groups. However, further works are essential to get better results.

An accumulating body of evidence demonstrates that a high dietary fiber intake is related to a lower risk of autoimmune diseases (22, 60). This protective effect of dietary fiber might be attributable to the immune-regulation properties of beneficial microbial metabolic products, such as SCFAs (61). Moreover, SCFAs induce multiple signaling pathways partly through their binding to G-protein coupled receptors (GPRs), particularly GPR41 and GPR43 (62). Studies have implicated a significant role for these GPRs in regulation of health and disease. SCFAs have been shown to have anti-inflammatory and antimicrobial effects, alter gut integrity and regulation of chemotaxis and phagocytosis (63–65). These findings highlight the role of SCFAs as a major signaling molecule that maintains the gut and immune homeostasis targeting metabolite sensing mechanisms such as GPRs. En-De Hu demonstrated that high-fiber diet and sodium butyrate can attenuate the development of AIH through regulation of immune regulatory cells and intestinal barrier function (22). Data from our study showed the concentration of SCFA, especially butyric acid, decreased in feces of AIH patients and B420 obviously increased the concentration of butyrate in cecal feces of EAH mice. However, the concentrations of isovaleric and valeric acid, which were significantly decreased in feces of AIH patients, were not decreased in EAH mouse model. The difficulty of animal models to fully replicate the pathophysiology of human diseases, and differences in the gut microbiota and dietary patterns between mice and humans may contribute to these differences. The underlying mechanisms of the SCFAs and liver immune homeostasis needs further study.

In the past two decades, the immunoregulatory effects of probiotic strains on innate and adaptive immune cells have been evaluated (66). Innate immune cells, for instance macrophages and dendritic cells, recognize microbes and respond to pathogen associated molecular patterns when the bacteria or metabolites are translocated across the intestinal barrier (67, 68). The activated macrophages secrete cytokines and chemokines, affect T-cell proliferation and differentiation and induce adaptive immune responses. Studies have reported that gut microbiota play an important role in shaping the Treg/Th17 axis in adaptive immune response (69). Alba et al. demonstrated the efficacy of Bifidobacterium for the treatment of patients with cirrhosis through inducing a morphologic, phenotypic and functional transition towards an anti-inflammatory profile (70). Rui Yu et al. investigated two *Bifidobacterium. adolescentis* strains for specific immunoregulatory effects, including protection of the Treg/Th17 axis of the cellular immune response system (71). Further studies show that cell surface polysaccharides of Bifidobacterium bifidum can induce the generation of Foxp3+ regulatory T cells through a partially Toll-like receptor 2-mediated mechanism (72). However, A recent study showed an association between high adhesion to epithelial cells of Bifidobacterium and Th17 cell induction, and a subsequent study identified *B. adolescentis* L2-32 as the first human-source commensal inducing Th17 cells (73, 74). Therefore, investigations of the immunoregulatory properties and molecular mechanisms that are critical for the specific function of these strains are of great importance.

In recent years, great importance has been attached to the role of intestinal bacteria in the pathogenesis of autoimmune diseases. It’s reported that translocation of intestinal pathobiont drives autoimmunity in mice and human and early but not late antibiotic treatment prevented chronic liver inflammation and autoantibodies (75, 76). Generally, patients with active diseases requires prednisone and immunosuppressive agents to control inflammation in the liver. In our study, early intervention with B420 could ameliorate liver injury of EAH mice and the mechanism involving regulating RIP3 signaling pathway of liver macrophage and cytokines profiles, thus impaired the differentiation of Th17 cells. Of note is that the inhibition effect of B420 on RIP3 signaling was blunted *in vitro* studies, indicating the key determinant factor of B420 on liver immune homeostasis is attributed to the regulation of intestinal barrier function. To our knowledge, our study is the first to focus on the effect of probiotics on regulating gut–liver axis in AIH. The novel finding of our study is that B420 can strengthen intestinal barrier function and further mitigate translocation of bacteria and their metabolites such as LPS, which have been implicated in inhibiting liver inflammation, thus significantly alleviate hepatitis caused by autoimmune factors. The dysregulation of RIP3 signaling is considered a crucial event inducing inflammation through necroptosis (30). Previous studies have already shown that LPS induced programmed cell death and thereby increased the expressions of its target genes, such as RIP3 and MLKL (77, 78). Recent studies demonstrated that some harmful pathogen also activated RIP3 signaling pathway including *Staphylococcus aureus*, *Chlamydia muridarum* and influenza H7N9 virus (79–81). In our study, mice in EAH group showed elevated serum LPS level and increased abundance of pathogenic bacteria including Bacteroides and Ruminococcus. Importantly, the activation of RIP3 signaling was inhibited by B420 treatment. Taken together, these findings demonstrate probiotics can alter the microbial composition of EAH mice and thus facilitate the maintenance of liver immune homeostasis. However, the molecular mechanisms by which probiotics regulate the immune response of the liver need to be further studied.

In summary, our findings showed that early intervention with B420 in EAH mice has beneficial functions and the underlying mechanisms involving modulating the gut microbiota composition and intestinal barrier function, inhibiting the RIP3 signaling pathway of liver macrophages thus decreasing the proportion of Th17 cells were deciphered. Our research shed light on the therapeutic and research potentials for the application of probiotics in AIH. However, the limitation of our study is the lack of evidence from clinical patients and the underlying mechanism targeting intestinal microbiota and intestinal barrier in AIH needs further study. Nevertheless, the encouraging results seen in the EAH model will surely promote further clinical trial development. Furthermore, application of probiotics might be novel options for treatment of AIH.

## Data Availability Statement

The 16S rRNA gene sequencing data has been uploaded to SRA—The BioProject ID is PRJNA657497.

## Ethics Statement

The studies involving human participants were reviewed and approved by the Ethical Committee of Tianjin Medical University General Hospital. This study didn't use client owned animals (noncommercially available animals e.g. pets or livestock). The patients/participants provided their written informed consent to participate in this study. The animal study was reviewed and approved by Authorization of the Ethical Committee of Tianjin Medical University General Hospital.

## Author Contributions

LZ and BW designed the study. HZ, ML, XL, and WZ performed the experiments. YL, YR, JZ, LG, and XC analyzed the results. HZ and LZ wrote the paper. HZ, ML, and XL contributed equally to this work. All authors contributed to the article and approved the submitted version.

## Funding

This study was supported by the grants (81860109 and 81470834) from the National Natural Science Foundation of China.

## Conflict of Interest

The authors declare that the research was conducted in the absence of any commercial or financial relationships that could be construed as a potential conflict of interest.
