# Supplementary material for: Bifidobacterium animalis ssp. Lactis 420 Mitigates Autoimmune Hepatitis Through Regulating Intestinal Barrier and Liver Immune Cells
Source: Front Immunol. 2020 Oct 6;11:569104. doi: 10.3389/fimmu.2020.569104 (PMC7573389; doi:10.3389/fimmu.2020.569104)
Supplement: Supplementary file 1 [file DataSheet_1.docx]

Supplementary Material


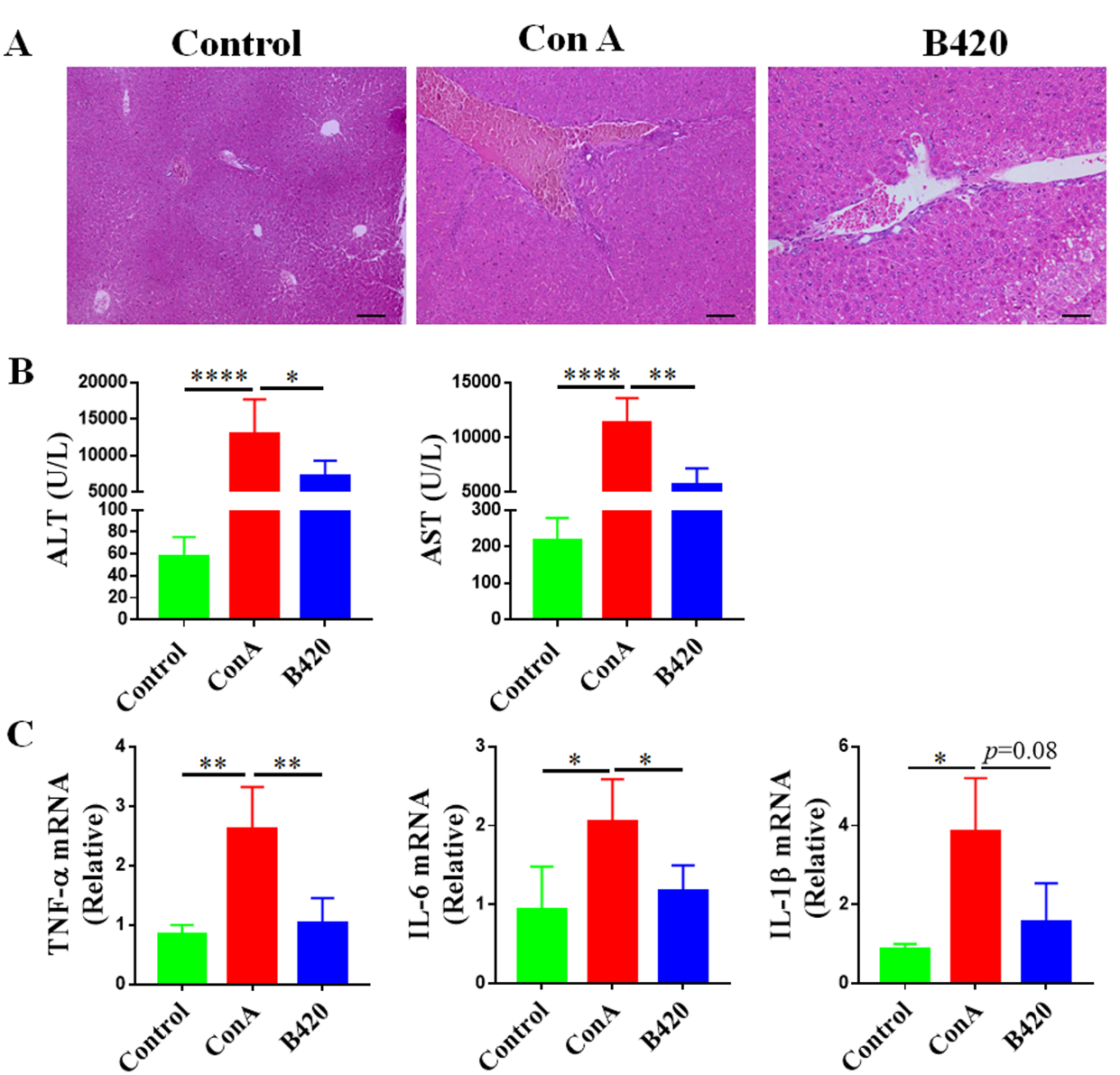


**Supplementary Figure 1.** B420 attenuated liver injury in Con A-mediated autoimmune hepatitis model. (A)Representative H&E images of liver tissues were shown. (B)The serum concentrations of ALT and AST were assessed. (C)The relative mRNA expressions of TNF-α, IL-6 and IL-1β in liver tissues. In (A–C), n=6 in each group. Scale bar: 50μm. The data were presented as means ± SD (Student’s t-test, *p < .05, **p < .01, ****p < .0001).


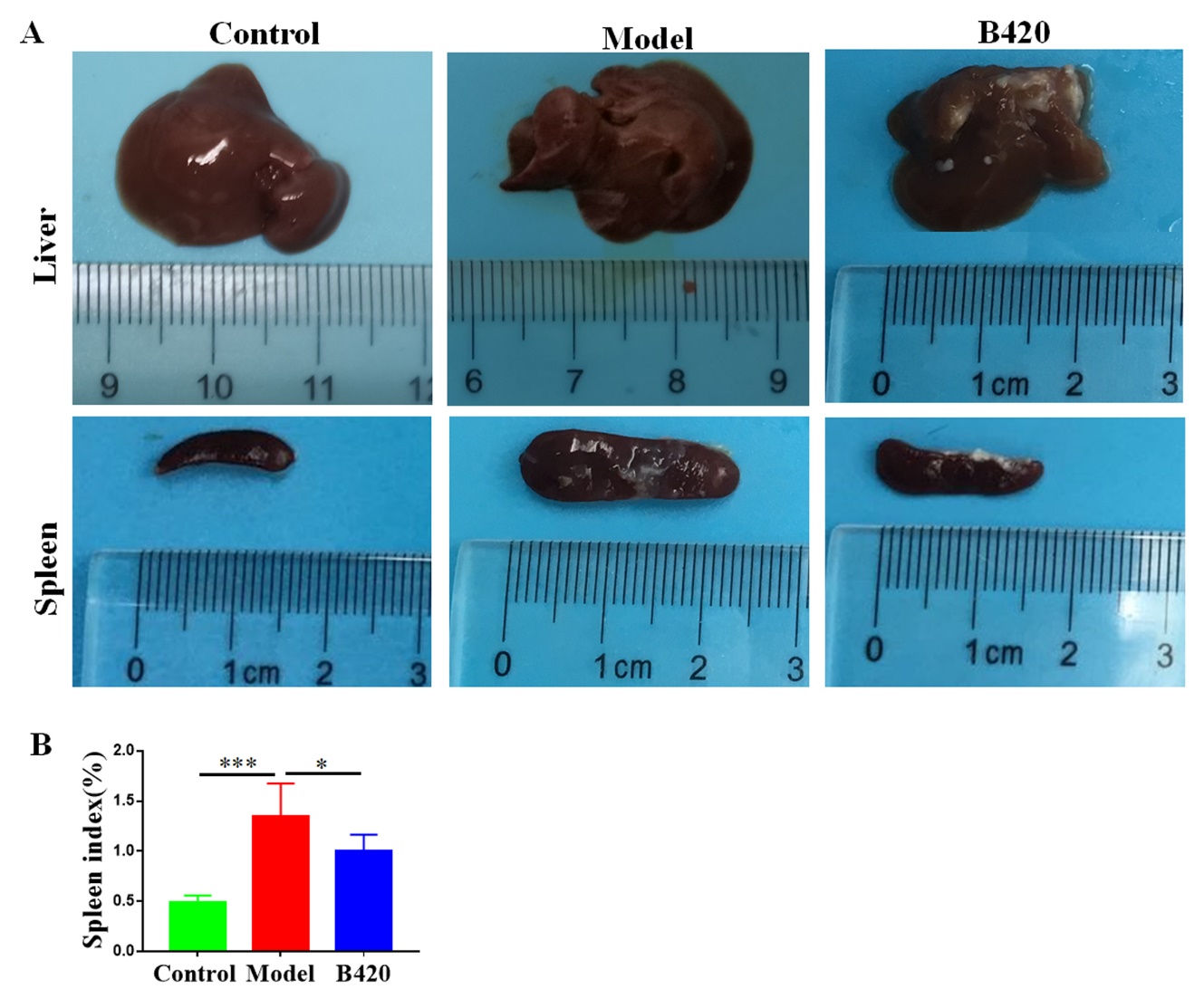


**Supplementary Figure 2.** The general view of liver and spleen and B420 decreased the spleen index. (A) The general view of liver and spleen of the three groups were shown. (B) The spleen index of the three groups were measured. In (A-B), n=6 in each group. The data were presented as means ± SD (Student’s t-test, *p < .005, ***p < .001).


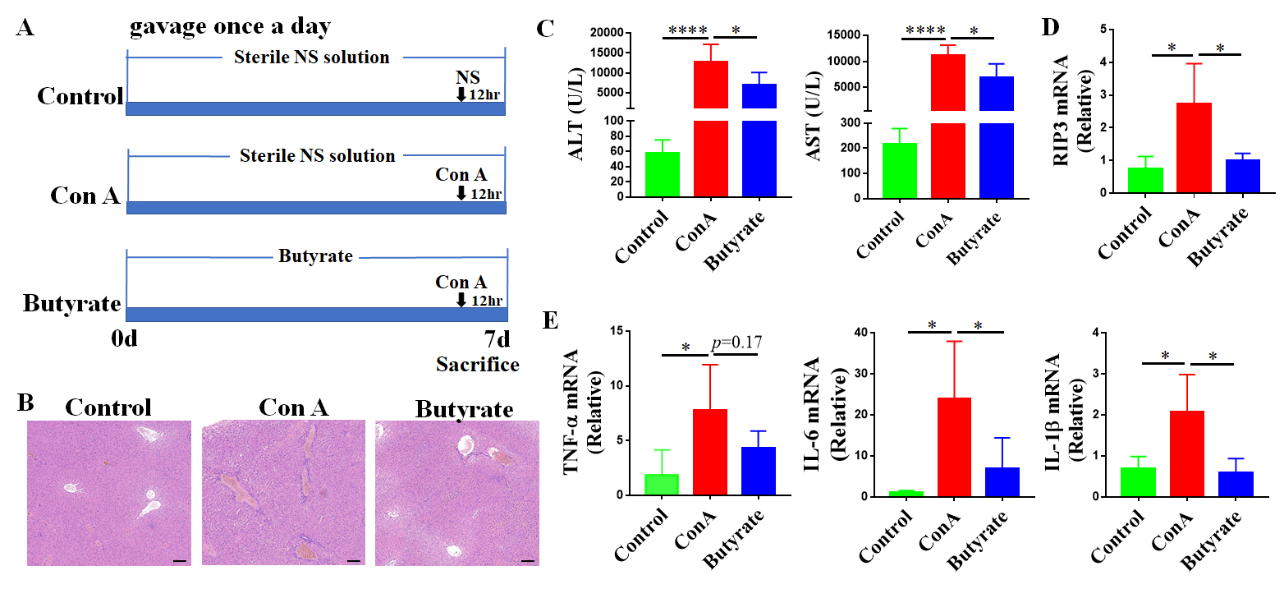


**Supplementary Figure 3.** Butyrate attenuated liver injury in EAH mice. (A)Modeling process of Con A-mediated autoimmune hepatitis model and administration of Butyrate. (B)Representative H&E images of liver tissues were shown (C)The serum concentrations of ALT and AST were assessed (D)The relative expression of RIP3 in liver tissues were analyzed by real-time PCR. (E)The relative mRNA expressions of TNF-α, IL-6 and IL-1β in liver tissues. In (A–E), n=6 in each group. Scale bar: 50μm.The data were presented as means ± SD (Student’s t-test, *p < .05, ****p < .0001).


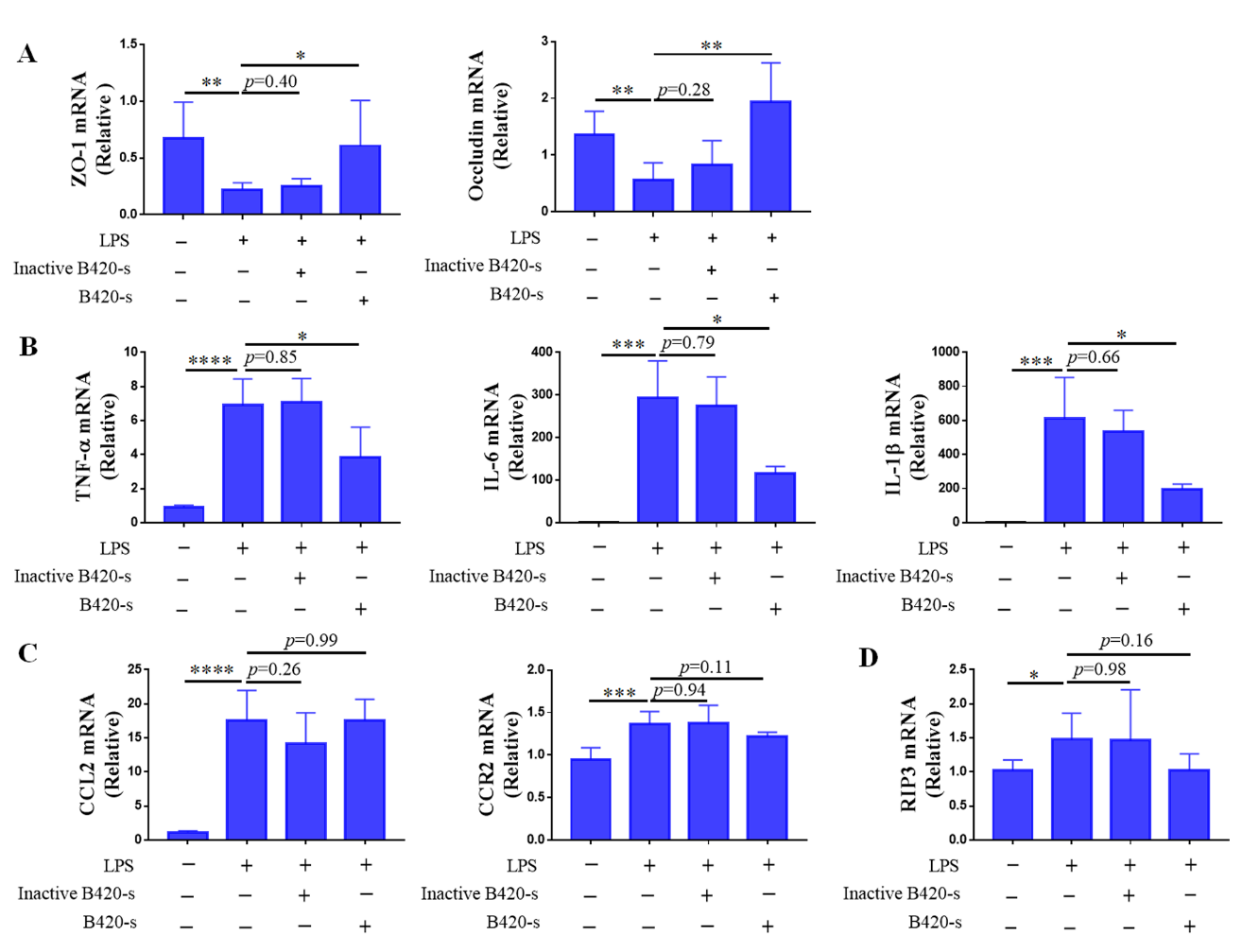


**Supplementary Figure 4.** Protective effects of B420-s in LPS-induced barrier injury of Caco-2 monolayers and activation of RAW264.7 cells. The cells were treated with LPS(3mg/ml) for 12h in the absence or presence of pre-treatment with inactive B420-s or B420-s. (A) The relative mRNA expressions of ZO-1 and Occludin in Caco-2 cells were analyzed. (B-D) TNF-α, IL-6 and IL-1β (B), CCL2 and CCR2 (C), RIP3 (D)mRNA levels in RAW24.7 cells were detected by quantitative real-time PCR. The data were presented as means ± SD of three independent experiments (Student’s t-test, *p < .05, **p < .01, ***p < .001, ****p < .0001).
